# *NPA*: an R package for computing network perturbation amplitudes using gene expression data and two-layer networks

**DOI:** 10.1186/s12859-019-3016-x

**Published:** 2019-09-03

**Authors:** Florian Martin, Sylvain Gubian, Marja Talikka, Julia Hoeng, Manuel C. Peitsch

**Affiliations:** PMI R&D, Philip Morris Products S.A, Quai Jeanrenaud 5, CH-2000 Neuchâtel, Switzerland

**Keywords:** Gene expression, Network models, Systems toxicology

## Abstract

**Background:**

High-throughput gene expression technologies provide complex datasets reflecting mechanisms perturbed in an experiment, typically in a treatment versus control design. Analysis of these information-rich data can be guided based on a priori knowledge, such as networks of related proteins or genes. Assessing the response of a specific mechanism and investigating its biological basis is extremely important in systems toxicology; as compounds or treatment need to be assessed with respect to a predefined set of key mechanisms that could lead to toxicity. Two-layer networks are suitable for this task, and a robust computational methodology specifically addressing those needs was previously published.

The *NPA* package (https://github.com/philipmorrisintl/NPA) implements the algorithm, and a data package of eight two-layer networks representing key mechanisms, such as xenobiotic metabolism, apoptosis, or epithelial immune innate activation, is provided.

**Results:**

Gene expression data from an animal study are analyzed using the package and its network models. The functionalities are implemented using R6 classes, making the use of the package seamless and intuitive. The various network responses are analyzed using the leading node analysis, and an overall perturbation, called the Biological Impact Factor, is computed.

**Conclusions:**

The *NPA* package implements the published network perturbation amplitude methodology and provides a set of two-layer networks encoded in the Biological Expression Language.

## Background

Gene expression technologies provide complex datasets reflecting the response of a cell system or organism exposed to bioactive substances. Contextualizing and quantifying the transcriptomic profiles into predefined mechanisms by combining the gene expression changes and networks is at the core of systems toxicology, as this discipline requires a quantitative measure of dose-response. Among the networks used, cause-and-effect network models are becoming increasingly popular [3, 7–9, 12, 13, 19, 25, 29–31, 34, 35], and many of these models, describing processes involved in cell proliferation, cell fate, cell stress, and inflammation, have already been published in repositories such as the Causal Bionet (http://causalbionet.com) [3] or BEL Commons (https://bel-commons.scai.fraunhofer.de) [14]. The underlying biological knowledge in these networks has been extracted from the scientific literature manually and encoded in the Biological Expression Language (BEL), a computable language developed specifically for causal biological networks (CBN) (http://bel.bio/).

To address the quantification of biological mechanisms known to be linked or that can lead to toxicological responses based on gene expression profiles, two-layer networks are suitable, and a mathematically and statistically sound methodology, called network perturbation amplitude (NPA), was published in [23]. The two-layer structure on the networks reflects backward reasoning, paradigm in which protein activities in a pathway are inferred from their downstream gene expression footprints, as opposed to forward reasoning that maps gene expression (or related fold-change) to protein activities. In this context, several studies using this approach have been published in recent years [10–13, 15, 16, 20, 21, 24, 26, 27, 36]. This approach is a threshold-free approach and can therefore enable an objective evaluation of network perturbations.

### Implementation

The *NPA* package has been developed in and designed for the R statistical environment. It is accessible in Bioconductor and free of charge for non-commercial use.

The implementation uses R6 classes with an S4-class dispatching in order to make its usage more intuitive. It does not rely on any C++ component, as the R code has been optimized to provide fast permutation tests (less than 5 s per two-layer structured network).

### Workflow overview

The quantification of NPAs aims to describe the response of biological mechanisms modeled by a network using transcriptomic data. Here we focus on the particular type of causal networks for which the NPA methodology has been developed. Given a suitably organized collection of causal networks selected for a priori relevant biological mechanisms, the structure of the associated NPA results can be seen as a complex reduction scheme starting from large experimental transcriptomic data. It provides a quantification of the treatment-induced impact on the considered biological mechanisms, which, in concrete applications, is used to comparatively assess toxicity.

**Fig. 1:**
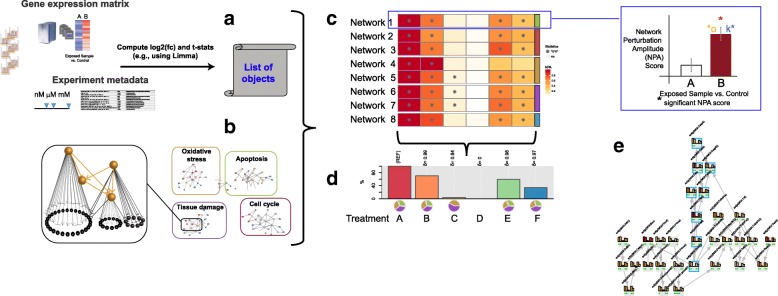
The NPA workflow. The gene expression data are used to estimate the treatment effect for each gene. The (log_2_) fold-changes and the associated *t*-statistics are required (**a**). By combining the gene expression fold-changes and a two-layer causal network (**b**), its perturbation is quantified and assessed for its significance by combining two specificity statistics and the fold-change standard deviations (**c**). Several NPAs can be summarized into a holistic quantity describing the overall biological response, called the Biological Impact Factor (BIF) (**d**), which can be used as a toxicological index for comparing various treatments. If an individual network reaches significance, the leading node analysis (**e**) enables the identification of the key biological entities involved in its response to ease the interpretation

Concretely, the workflow of the NPA computational workflow requires three distinct inputs in terms of experimental data and biological knowledge (Fig. 1).

### Network input

The network models provided with the companion package, *NPAModels* (https://github.com/philipmorrisintl/NPAModels), represent the molecular mechanisms across wide range of biological processes, including cell fate, cell stress, cell proliferation, inflammation, and tissue remodeling, relevant for human respiratory physiology. The two-layer structure, as defined in [23], is summarized below.

#### Network functional layer

Unlike other types of networks, the networks nodes describe molecular (protein, chemicals, genes) concentrations but also represent functions such as transcriptional, enzymatic, or kinase activities. The network edges encode directed relationships between nodes, each of which is associated with a sign representing the increasing or decreasing direction of change between the molecular activities. The biological knowledge represented in these networks has been manually extracted from the scientific literature and encoded in the BEL, an ontology developed specifically for CBNs (http://www.openbel.org/). More CBNs and BEL resources are publicly and freely available on the CBN Database website (http://causalbionet.com) [3].

#### Network transcript layer

For some nodes in the functional layer, information supporting the relationship to the expression (upregulation or downregulation) of certain genes is available. These relationships are either extracted from literature or from specific gene expression experiments. Such a footprint for a given node in the functional layer (such as a transcription factor or an activity of a protein) can be efficiently extracted from gene expression data sets whereby the experiment involves the inhibition or activation of the molecule under consideration. Edges from that node to genes significantly impacted are defined and signed accordingly. These specific edges (directed and signed) define the gene expression fingerprint of the network functional layer. Typically, hundreds or thousands of genes are included in the transcript layer. Note that not all the nodes are required to have such footprints for computing NPA. Following the “backward reasoning”, paradigm stating that changes in molecular mechanisms encoded by causal network nodes (e.g., the activity of a transcription factor) can be deduced from the expression changes of their downstream-regulated genes, a gene expression profile can be used to computationally predict the activity of the functional layer nodes.

### Data input

The gene expression profiles are derived from gene expression data for which one or several contrasts (typically a treatment versus control design, or linear model coefficients) are estimated from a statistical model. These contrasts represent the system response profile associated with comparisons of interest and are mapped onto the transcript layer of the two-layer network, which reflects the perturbation of the functional layer. In our implementation, the data input is a list, one entry per contrast, and each entry is a data frame, with a variable *nodeLabel* describing the gene symbol of each row, *foldChange* describing the estimated contrast (log_2_-based), and *t* describing the *t*-statistics associated with the fold-change. *T*-statistics are typically derived from *limma* (in which case the moderated *t*-statistics is used) or from *DESeq2* for RNA-seq data (in which case the *t*-statistics is replaced by the *z*-statistics of the Wald test used for testing the significance of the shrunken estimates of the gene fold-changes).

### NPA computation

The NPA method previously reported in [12, 13, 23] will first compute the perturbations for the network nodes based on a constraint optimization problem. If the obtained transcriptomics profile reflects the perturbation of the functional layer, all of the differential values should be close to each other (smooth perturbation profile) while being equal to the observed fold-changes β in the transcript layer, denoted V_0_. The differential values for the functional layer are obtained by solving:

$$ {\left.{\mathit{\min}}_{f\in {l}^2(V)}{\sum}_{x\to y}{\left(f(x)-\sigma \left(x\to y\right)\cdotp f(y)\right)}^2\ s.t.\kern0.5em f\right|}_{V_0}=\beta $$

where *σ*(*x* → *y*) denotes the sign of the edge *x* → *y*. This constrained optimization problem can be solved analytically and was implemented as a matrix multiplication, relying on sub-matrices of the signed Laplacian matrix of the two layers [23]. Once differential values are obtained for the functional layer, the NPA score is then computed by summing the result over the edges of that layer as:

$$ NPA=\frac{1}{\mid E\mid}\sum \limits_{e\  in\ E}{\left(f\left({e}_0\right)+\sigma (e)f\left({e}_1\right)\right)}^2 $$

where E is the set of its edges, |E| is its size, f is the solution of the constrained problem describing the node differential values, and e_0_ and e_1_ denote the start and the end, respectively, of the edge e. This sum is efficiently implemented as the evaluation of a quadratic form.

Finally, the computation of three accompanying statistics is performed to determine if the obtained NPA value represents a true positive. The first statistic is based on the biological variability propagated from the uncertainties of the differential gene expression values: the 95% confidence interval (CI) around the NPA value should not contain zero. The other two statistics test the relevance of the biological mechanism(s) encoded in the network by randomly reshuffling the network edges of the transcriptional layer or functional layer. This turns into two null distributions for the network-level perturbation values. If the actual NPA value lies above the 95% quantile of a null distribution, it is considered to be statistically significant and labeled as “O” or “K,” respectively. Significant network perturbations correspond to the cases where all three statistical tests lead to significance.

In our implementation, computation time for those statistics usually takes 49.1 s for 12 comparisons and 58.1 s for 48 comparisons and scales linearly with the number of comparisons by using the precomputed permutation matrices. To speed up the computation, it is recommended to precompute the permutation matrices using the function *preprocessNetworks()* provided by the *NPAModels* companion package. With the preprocessed version of the networks, the computation time drastically drops to 6.4 s for 12 comparisons and 20.1 s for 48 comparisons, on single Intel® Core™ i5–6500 CPU @ 3.20GHz with a 16 Gb of RAM computer. If a node of the functional layer has less than five edges to the transcript layer (considered as under-represented), removing those edges is recommended. Finally, only the nodes having an ancestor and a descendant node with edges to the transcript layer are scored to avoid extrapolation.

All of the computations are performed with a single call of *compute npa* and plotted using the *barplot* method. Eight two-layer networks are available in the accompanying *NPAModels* package *.*

The interpretation of a significant network perturbation is supported by the inspection of the leading nodes, defined as the functional layer nodes that contribute the most to the NPA scores. This component is key, as two different perturbation patterns in the functional layer can lead to the same summarized scores and as functional layer size can be substantial (e.g., oxidative stress network functional layer contains 194 nodes) making the systematic inspection of all differential values tedious. Leading nodes are nodes of the functional layer, whose differential values contribute 80% to the observed effect. The inspection of this shorter list of nodes supports the interpretation and the comparison of network perturbation across contrasts. It also allows for the assessment of the directionality (activation or inhibition) of the inferred effects on each node; as the NPA is by definition positive. Leading node ranks and signs can be accessed with the *as.matrix* method from an NPA object and visualized as a heatmap or in the network graph by calling the *plot* method.

To further ease the interpretation and identification of highly impacted sub-networks, network sub-graphs (called here modules) that are dense in leading nodes across all the contrasts can be extracted by the method *module* and plotted by a simple *plot* call. The modules are extracted by using the *dNetFind* call from the *dnet* package in order to heuristically find a maximum scoring connected subgraph, using the leading node contributions as scores. If modules are too large for a visual inspection, they can be further clustered by using the *infomap* community approach implemented in the *igraph* package.

When several networks are available and grouped into families (such as Cell Stress, Cell Fate), the treatment-induced biological impact is summarized as network family-level impact factors, which in turn are summarized into a single quantity, called the BIF. The evaluation of the BIF consists of filtering out the networks that are not significantly perturbed and then summing the remaining NPA values with weights that take into account the number of network in each family and the nodes overlapping between networks.

## Results

### Transcriptomic dataset

**Fig. 2:**
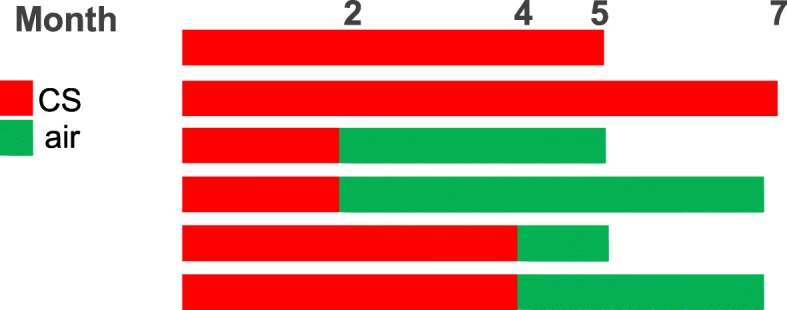
E-MTAB-2756. Study design and time points, from which the transcriptomic were used

To show the scoring of the network models with transcriptomic data, we have focused on a mouse inhalation study reported in [4]. The study was designed to identify the onset of emphysema induced by exposure to cigarette smoke (CS). C57BL/6 mice were exposed to mainstream CS from the 3R4F reference cigarette through whole-body exposure for up to 7 months. Additionally, four cessation scenarios were included to assess the impact of smoking cessation on emphysema progression in the lung. Figure 2 illustrates the study arms used in this analysis. The dataset is available in the package as data (COPD1).

### NPA and BIF

**Fig. 3:**
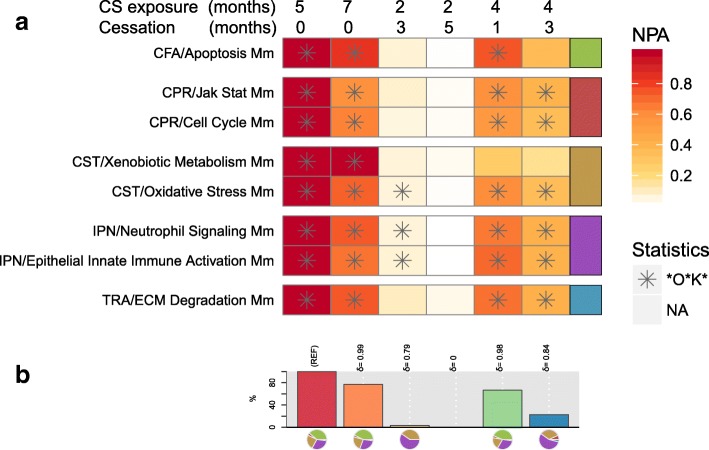
The NPA and the BIF. **a** Heatmap of the NPA scores. A network is considered perturbed if, in addition to the significance of the NPA score with respect to the experimental variation, the two companion statistics (O and K), derived to inform on the specificity of the NPA score with respect to the biology described in the network, are significant. *O and K statistic *p*-values below 0.05 and NPA significant with respect to the experimental variation. **b** BIF scores. The percentages give the relative biological impact, which is derived from the cumulated network perturbations caused by the treatment relative to the reference (the treatment showing the highest perturbation; here 3R4F as REF). Only the significant network perturbations are summarized further into this single number; hence, any component entering the score is significant by definition. The contribution to the score for each network family is indicated by a pie chart underneath each bar. For each treatment comparison, the δ value (− 1 to 1) indicates how similar the underlying network perturbations are with respect to the reference. A value of 1 indicates that all the networks are perturbed by the same mechanisms

The NPA scores, describing the degree perturbation of each network as a positive amplitude, are shown in Fig. 3a. To generate NPA scores on the whole network suite and displaying the results, the following code was used:

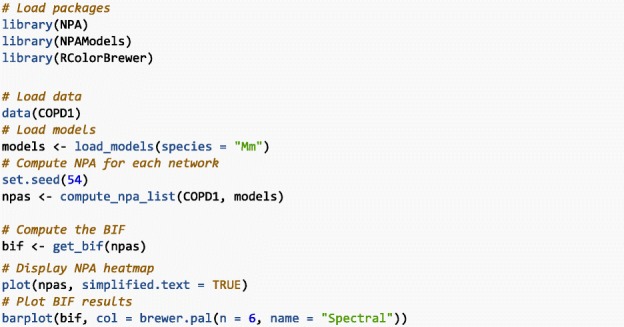



All network models were impacted in response to 5 and 7 months of CS exposure. These impacts were remarkably reduced in the lungs of mice that were exposed to fresh air after 2 months of CS exposure and to a lesser extent when the CS exposure was stopped after 4 months. A similar result was seen in the BIF that depicts the aggregated impact based on all networks that were scored with the transcriptomic data (Fig. 3b).

### Leading node analysis

**Fig. 4:**
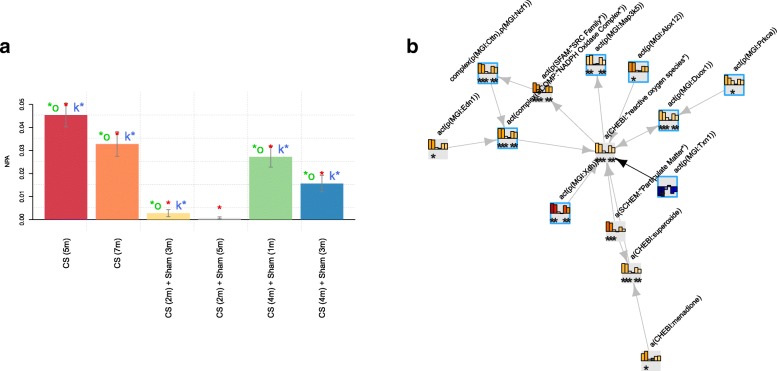
NPA of the oxidative stress network model scored with E-MTAB-2756 transcriptomic data. The bar graph on the left (panel **a**) represents the perturbation amplitude of the network model as a whole, and the network presentation on the right (panel **b**) shows an example of connected leading nodes common to all contrasts. The backbone NPA values with directionalities of inferred regulation are shown as bar graphs for each node. The green asterisk indicates that the node is a leading node in a given contrast. Highlighted nodes are the molecules discussed in the main text. The vocabulary for the BEL is provided in http://www.openbel.org/

The leading node analysis was used to dissect the mechanistic detail behind the perturbation of each network model in response to CS exposure. To investigate the network perturbation, a graphical presentation of the leading node module in the oxidative stress network model was leveraged (Fig. 4) and generated using the code below:

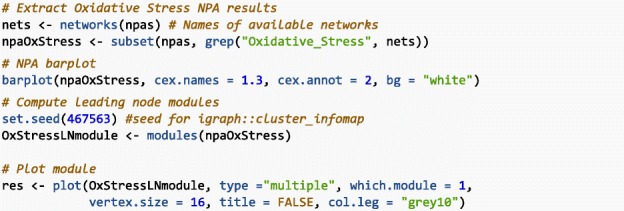



The graph illustrates the signaling cascade from particulate matter exposure to the increase in superoxide production, leading to increased intracellular reactive oxygen species (ROS), which also results from the activation of the arachidonate 12-lipoxygenase (ALOX12) [1] and xanthine dehydrogenase (XDH) [33]. In contrast, thioredoxin (TXN) was inferred to be inhibited, in line with its role in modulating intracellular ROS balance [18]. The increase in ROS triggers multiple signaling pathways, such as the SRC family leading to activation of the NADPH oxidase complex via cortactin (CTTN) and neutrophil cytosolic factor 1 (NCF1) [32]. The NADPH oxidase complex in turn further increases the production of ROS [2]. Other signaling molecules predicted to be activated in response to CS and the increase in ROS in the mouse lung include Duox1 and Map3k5.

**Fig. 5:**
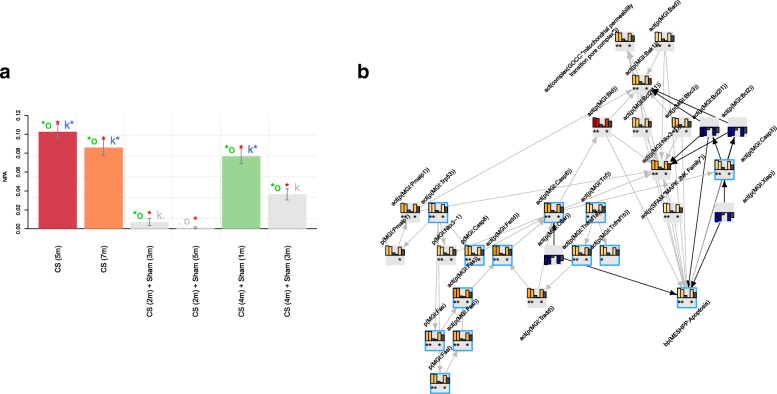
NPA of the apoptosis network model scored with E-MTAB-2756 transcriptomic data. The bar graph (panel **a**) on the left represents the perturbation amplitude of the network model as a whole shown with the CIs accounting for experimental variation. The red star indicates that the score is statistically different from 0. In addition, companion statistics derived to inform on the specificity of the score with respect to the network structure are shown as *O and *K, respectively, if their *p*-values are below the significance level of 0.05, and by .O and K. when the corresponding *p*-values are between 0.05 and 0.1. Symbol legend: *: O and K statistic *p*-values below 0.05 (in color), .O and K. *p*-values between 0.05 and 0.1 (in grey). The network presentation on the right (panel **b**) shows an example of connected leading nodes common to all contrasts. The backbone NPA values with directionalities of inferred regulation are shown as bar graphs for each node. The green asterisk indicates that the node is a leading node. Highlighted nodes are the molecules discussed in the main text. The vocabulary for the BEL is provided in http://www.openbel.org/

The apoptosis network model represents one of the cell fates that can result from prolonged oxidative stress and tissue damage. Figure 5 shows the perturbation for the apoptosis network as a whole and the leading nodes inferred from the transcriptomic data from CS-exposed mouse lungs. The activation of caspase-8 mediated by p53 and the FAS/FADD pathway was inferred from the transcriptomic data, in line with the CS toxicants, such as acrolein, activating apoptosis pathways in lung cells [28]. TNFα pathway in the context of apoptosis was also inferred to mediate caspase activation in this dataset.

**Fig. 6:**
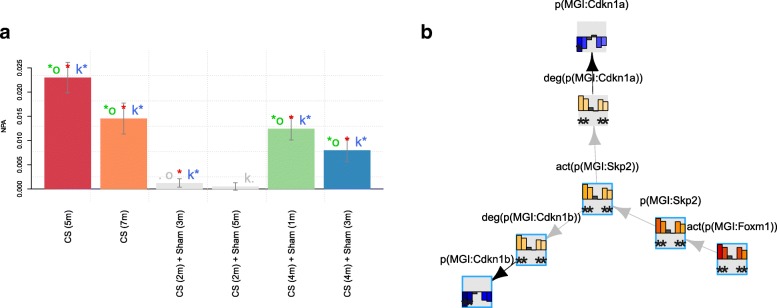
NPA of the cell cycle network model scored with E-MTAB-2756 transcriptomic data. The bar graph on the left (panel **a**) represents the perturbation amplitude of the network model as a whole shown with the CIs accounting for experimental variation. The red star indicates that the score is statistically different from 0. In addition, companion statistics derived to inform on the specificity of the score with respect to the network structure are shown as *O and K*, respectively, if their *p*-values are below the significance level of 0.05, and by .O and K. when the corresponding *p*-values are between 0.05 and 0.1. Symbol legend: *: O and K statistic *p*-values below 0.05 (in color), .O and K. *p*-values between 0.05 and 0.1 (in grey). The network presentation on the right (panel **b**) shows an example of connected leading nodes common to all contrasts. The backbone NPA values with directionalities of inferred regulation are shown as bar graphs for each node. The green asterisk indicates that the node is a leading node in a given contrast. Highlighted nodes are the molecules discussed in the main text. The vocabulary for the BEL is provided in http://www.openbel.org/

Inferred activation of FOXM1, subsequent activation of SKP2, and the degradation of cyclin-dependent kinase inhibitor 1B (CDKN1b) were observed based on the leading node analysis of the cell cycle network scored with transcriptomic data from the lungs of CS-treated mice [5] (Fig. 6).

**Fig. 7:**
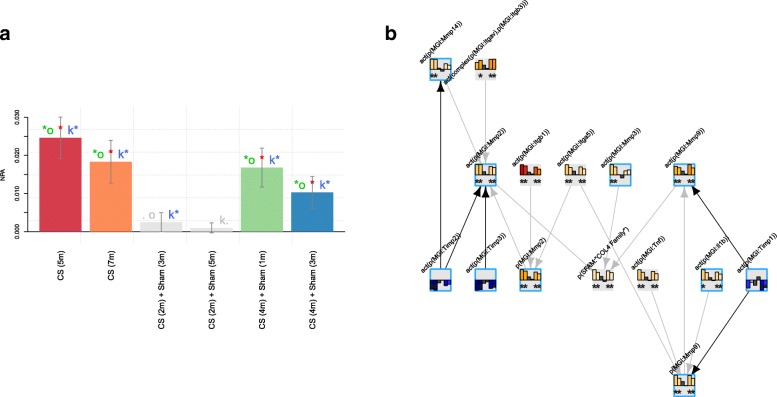
NPA of the ECM degradation network model scored with E-MTAB-2756 transcriptomic data. The bar graph on the left (panel **a**) represents the perturbation amplitude of the network model as a whole shown with the CIs accounting for experimental variation. The red star indicates that the score is statistically different from 0. In addition, companion statistics derived to inform on the specificity of the score with respect to the network structure are shown as *O and K*, respectively, if their *p*-values are below the significance level of 0.05, and by .O and K. when the corresponding *p*-values are between 0.05 and 0.1. Symbol legend: *: O and K statistic *p*-values below 0.05 (in color), .O and K. *p*-values between 0.05 and 0.1(in grey). The network presentation on the right (panel **b**) shows an example of connected leading nodes common to all contrasts. The backbone NPA values with directionalities of inferred regulation are shown as bar graphs for each node. The green asterisk indicates that the node is a leading node. Highlighted nodes are the molecules discussed in the main text. The vocabulary for the BEL is provided in http://www.openbel.org/

The degradation of the extracellular matrix (ECM) is characteristic of emphysema, which is a manifestation of chronic obstructive pulmonary disease and a phenotype detected in the lungs of mice exposed to CS [6, 22]. The graphical presentation of the leading nodes that are connected in the ECM degradation network model is shown in Fig. 7. The tissue inhibitor of matrix metalloproteases (TIMP) were inferred to be downregulated, and accordingly, the matrix metalloproteases (MMP) were inferred to be activated, with subsequent degradation of collagen in the lungs of mice exposed to CS. The leading node analysis also indicated that increased IL1b signaling was responsible for the molecular cascade leading to the degradation of the ECM components.

## Conclusions

The *NPA* package and its companion data package *NPAModels* provide the tools and resources to contextualize transcriptomic profiles into manually curated networks describing cellular processes relevant to toxicology, such as apoptosis, cell cycle, oxidative stress, and phase I xenobiotic metabolism. This network enrichment approach [23] can be considered as a threshold-free functional class scoring methodology [17]. The computations and presentations of the results have been facilitated by using R6 classes and provides the user with an intuitive and easy-to-use package.

## Data Availability

GitHub project page: https://github.com/philipmorrisintl/NPA; submitted to Bioconductor. The R packages *NPA* and *NPAModels* are both submitted to the Bioconductor project http://www.bioconductor.org and require an R version greater than 3.5.1.

## Abbreviations

ALOX12: Arachidonate 12-lipoxygenase
BEL: Biological Expression Language
BIF: Biological Impact Factor
CBN: Causal Biological Network
CDKN1b: Cyclin-dependent kinase inhibitor 1B
CS: Cigarette Smoke
CTTN: Cortactin
ECM: Extracellular matrix
MMP: Matrix metalloproteases
NCF1: Neutrophil cytosolic factor 1
NPA: Network Perturbation Amplitude
ROS: Reactive Oxygen Species
TIMP: Tissue inhibitor of matrix metalloproteases
TXN: Thioredoxin
XDH: Xanthine dehydrogenase

## References

[1] Armstrong, D., and Stratton, R.D. Oxidative stress and antioxidant protection: the science of free radical biology and disease 2016 Wiley. New York.

[2] Babior BM (2004). NADPH oxidase. Curr Opin Immunol.

[3] Boué, S., Talikka, M., Westra, J.W., Hayes, W., Di Fabio, A., Park, J., Schlage, W.K., Sewer, A., Fields, B., and Ansari, S. Causal biological network database: a comprehensive platform of causal biological network models focused on the pulmonary and vascular systems Database 2015;2015. 10.1093/database/bav030 PMC4401337 25887162

[4] Cabanski M, Fields B, Boue S, Boukharov N, DeLeon H, Dror N, Geertz M, Guedj E, Iskandar A, Kogel U (2015). Transcriptional profiling and targeted proteomics reveals common molecular changes associated with cigarette smoke-induced lung emphysema development in five susceptible mouse strains. Inflamm Res.

[5] Carrano AC, Eytan E, Hershko A, Pagano M (1999). SKP2 is required for ubiquitin-mediated degradation of the CDK inhibitor p27. Nat Cell Biol.

[6] D'Armiento J, Dalal SS, Okada Y, Berg RA, Chada K (1992). Collagenase expression in the lungs of transgenic mice causes pulmonary emphysema. Cell.

[7] De Leon H, Boue S, Schlage WK, Boukharov N, Westra JW, Gebel S, VanHooser A, Talikka M, Fields RB, Veljkovic E (2014). A vascular biology network model focused on inflammatory processes to investigate atherogenesis and plaque instability. J Transl Med.

[8] Domingo-Fernández D, Kodamullil AT, Iyappan A, Naz M, Emon MA, Raschka T, Karki R, Springstubbe S, Ebeling C, Hofmann-Apitius M (2017). Multimodal mechanistic signatures for neurodegenerative diseases (NeuroMMSig): a web server for mechanism enrichment. Bioinformatics.

[9] Gebel S, Lichtner RB, Frushour B, Schlage WK, Hoang V, Talikka M, Hengstermann A, Mathis C, Veljkovic E, Peck M (2013). Construction of a computable network model for DNA damage, autophagy, cell death, and senescence. Bioinf Biol Insights.

[10] Gonzalez-Suarez I, Martin F, Marescotti D, Guedj E, Acali S, Johne S, Dulize R, Baumer K, Peric D, Goedertier D (2015). In vitro systems toxicology assessment of a candidate modified risk tobacco product shows reduced toxicity compared to that of a conventional cigarette. Chem Res Toxicol.

[11] Gonzalez-Suarez I, Sewer A, Walker P, Mathis C, Ellis S, Woodhouse H, Guedj E, Dulize R, Marescotti D, Acali S (2014). Systems biology approach for evaluating the biological impact of environmental toxicants in vitro. Chem Res Toxicol.

[12] Hoeng J, Deehan R, Pratt D, Martin F, Sewer A, Thomson TM, Drubin DA, Waters CA, de Graaf D, Peitsch MC (2012). A network-based approach to quantifying the impact of biologically active substances. Drug Discov Today.

[13] Hoeng J, Talikka M, Martin F, Sewer A, Yang X, Iskandar A, Schlage WK, Peitsch MC (2013). Case study: the role of mechanistic network models in systems toxicology. Drug Discov Today.

[14] Hoyt CT, Domingo-Fernandez D, and Hofmann-Apitius M. BEL Commons: an environment for exploration and analysis of networks encoded in Biological Expression Language. Database. 2018. 10.1093/database/bay126 PMC6301338 30576488

[15] Iskandar AR, Gonzalez-Suarez I, Majeed S, Marescotti D, Sewer A, Xiang Y, Leroy P, Guedj E, Mathis C, Schaller J-P (2016). A framework for in vitro systems toxicology assessment of e-liquids. Toxicol Mech Methods.

[16] Iskandar Anita R., Martin Florian, Talikka Marja, Schlage Walter K., Kostadinova Radina, Mathis Carole, Hoeng Julia, Peitsch Manuel C. (2013). Systems Approaches Evaluating the Perturbation of Xenobiotic Metabolism in Response to Cigarette Smoke Exposure in Nasal and Bronchial Tissues. BioMed Research International.

[17] Khatri P, Sirota M, Butte AJ (2012). Ten years of pathway analysis: current approaches and outstanding challenges. PLoS Comput Biol.

[18] Kim A, Joseph S, Khan A, Epstein CJ, Sobel R, Huang T-T (2010). Enhanced expression of mitochondrial superoxide dismutase leads to prolonged in vivo cell cycle progression and up-regulation of mitochondrial thioredoxin. Free Radic Biol Med.

[19] Kodamullil AT, Younesi E, Naz M, Bagewadi S, Hofmann-Apitius M (2015). Computable cause-and-effect models of healthy and Alzheimer's disease states and their mechanistic differential analysis. Alzheimers Dement.

[20] Kogel U, Suarez IG, Xiang Y, Dossin E, Guy P, Mathis C, Marescotti D, Goedertier D, Martin F, Peitsch M (2015). Biological impact of cigarette smoke compared to an aerosol produced from a prototypic modified risk tobacco product on normal human bronchial epithelial cells. Toxicol in Vitro.

[21] Kogel U, Titz B, Schlage WK, Nury C, Martin F, Oviedo A, Lebrun S, Elamin A, Guedj E, Trivedi K (2016). Evaluation of the tobacco heating system 2.2. Part 7: systems toxicological assessment of a mentholated version revealed reduced cellular and molecular exposure effects compared with mentholated and non-mentholated cigarette smoke. Regul Toxicol Pharmacol.

[22] Mahadeva R, Shapiro S (2002). Chronic obstructive pulmonary disease• 3: experimental animal models of pulmonary emphysema. Thorax.

[23] Martin F, Sewer A, Talikka M, Xiang Y, Hoeng J, Peitsch MC (2014). Quantification of biological network perturbations for mechanistic insight and diagnostics using two-layer causal models. BMC Bioinformatics.

[24] Oviedo A, Lebrun S, Kogel U, Ho J, Tan WT, Titz B, Leroy P, Vuillaume G, Bera M, Martin F (2016). Evaluation of the tobacco heating system 2.2. Part 6: 90-day OECD 413 rat inhalation study with systems toxicology endpoints demonstrates reduced exposure effects of a mentholated version compared with mentholated and non-mentholated cigarette smoke. Regul Toxicol Pharmacol.

[25] Park J, Schlage W, Frushour B, Talikka M, Toedter G (2013). Construction of a computable network model of tissue repair and angiogenesis in the lung. J Clinic Toxicol S.

[26] Phillips B, Veljkovic E, Boué S, Schlage WK, Vuillaume G, Martin F, Titz B, Leroy P, Buettner A, Elamin A (2015). An 8-month systems toxicology inhalation/cessation study in Apoe−/− mice to investigate cardiovascular and respiratory exposure effects of a candidate modified risk tobacco product, THS 2.2, compared with conventional cigarettes. Toxicol Sci.

[27] Phillips B, Veljkovic E, Peck MJ, Buettner A, Elamin A, Guedj E, Vuillaume G, Ivanov NV, Martin F, Boué S (2015). A 7-month cigarette smoke inhalation study in C57BL/6 mice demonstrates reduced lung inflammation and emphysema following smoking cessation or aerosol exposure from a prototypic modified risk tobacco product. Food Chem Toxicol.

[28] Roy J, Pallepati P, Bettaieb A, Averill-Bates DA (2010). Acrolein induces apoptosis through the death receptor pathway in A549 lung cells: role of p53. Can J Physiol Pharmacol.

[29] Schlage WK, Westra JW, Gebel S, Catlett NL, Mathis C, Frushour BP, Hengstermann A, Van Hooser A, Poussin C, Wong B (2011). A computable cellular stress network model for non-diseased pulmonary and cardiovascular tissue. BMC Syst Biol.

[30] Szostak, J., Ansari, S., Madan, S., Fluck, J., Talikka, M., Iskandar, A., De Leon, H., Hofmann-Apitius, M., Peitsch, M.C., and Hoeng, J. Construction of biological networks from unstructured information based on a semi-automated curation workflow. Database 2015 2015. 10.1093/database/bav057 PMC5630939 26200752

[31] Talikka M, Bukharov N, Hayes WS, Hofmann-Apitius M, Alexopoulos L, Peitsch MC, Hoeng J (2017). Novel approaches to develop community-built biological network models for potential drug discovery. Expert Opin Drug Discovery.

[32] Usatyuk PV, Romer LH, He D, Parinandi NL, Kleinberg ME, Zhan S, Jacaobson JR, Dudek S, Pendyala S, Garcia JG. Regulation of hyperoxia-induced NADPH oxidase activation in human lung endothelial cells by the actin cytoskeleton and cortactin. J Biol Chem. 2007. 10.1074/jbc.M700535200 17562703

[33] Wang C-H, Zhang C, Xing X-H (2016). Xanthine dehydrogenase: an old enzyme with new knowledge and prospects. Bioengineered.

[34] Westra JW, Schlage WK, Frushour BP, Gebel S, Catlett NL, Han W, Eddy SF, Hengstermann A, Matthews AL, Mathis C (2011). Construction of a computable cell proliferation network focused on non-diseased lung cells. BMC Syst Biol.

[35] Westra JW, Schlage WK, Hengstermann A, Gebel S, Mathis C, Thomson T, Wong B, Hoang V, Veljkovic E, Peck M (2013). A modular cell-type focused inflammatory process network model for non-diseased pulmonary tissue. Bioinform Biol Insights.

[36] Zanetti F, Sewer A, Mathis C, Iskandar AR, Kostadinova R, Schlage WK, Leroy P, Majeed S, Guedj E, Trivedi K (2016). Systems toxicology assessment of the biological impact of a candidate modified risk tobacco product on human organotypic oral epithelial cultures. Chem Res Toxicol.

